# On the Gas Storage Properties of 3D Porous Carbons Derived from Hyper-Crosslinked Polymers

**DOI:** 10.3390/polym11040588

**Published:** 2019-04-01

**Authors:** Giorgio Gatti, Mina Errahali, Lorenzo Tei, Maurizio Cossi, Leonardo Marchese

**Affiliations:** Dipartimento di Scienze e Innovazione Tecnologica, Università del Piemonte Orientale “A. Avogadro”, Via T. Michel 11, 15121 Alessandria, Italy; mina.errahali@uniupo.it (M.E.); lorenzo.tei@uniupo.it (L.T.); maurizio.cossi@uniupo.it (M.C.); leonardo.marchese@uniupo.it (L.M.)

**Keywords:** activated carbon, hyper-crosslinked polymers, gas storage, raman spectroscopy

## Abstract

The preparation of porous carbons by post-synthesis treatment of hypercrosslinked polymers is described, with a careful physico-chemical characterization, to obtain new materials for gas storage and separation. Different procedures, based on chemical and thermal activations, are considered; they include thermal treatment at 380 °C, and chemical activation with KOH followed by thermal treatment at 750 or 800 °C; the resulting materials are carefully characterized in their structural and textural properties. The thermal treatment at temperature below decomposition (380 °C) maintains the polymer structure, removing the side-products of the polymerization entrapped in the pores and improving the textural properties. On the other hand, the carbonization leads to a different material, enhancing both surface area and total pore volume—the textural properties of the final porous carbons are affected by the activation procedure and by the starting polymer. Different chemical activation methods and temperatures lead to different carbons with BET surface area ranging between 2318 and 2975 m^2^/g and pore volume up to 1.30 cc/g. The wise choice of the carbonization treatment allows the final textural properties to be finely tuned by increasing either the narrow pore fraction or the micro- and mesoporous volume. High pressure gas adsorption measurements of methane, hydrogen, and carbon dioxide of the most promising material are investigated, and the storage capacity for methane is measured and discussed.

## 1. Introduction

In the current hydrocarbon economy, transportation is fueled primarily by petroleum. The burning of hydrocarbon fuels has an adverse effect on the environment, as it is responsible for the increase of CO_2_ and other pollutants. A number of potential solutions for conservation and remediation of the environment threatened by CO_2_ emission growth are cutting edge research topics. These include studies on CO_2_ capture and storage [1], as well as on the use of cleaner fuels, such as natural gas (mostly CH_4_) [2] or hydrogen (H_2_) [3,4].

New porous materials characterized by appropriate surface area and nanometer pore size have been developed both for an optimal gas storage [5,6,7,8] and for applications in different fields, such as molecular separations [9,10,11] and catalysis [12,13,14]. Microporous materials, such as activated carbons (ACs) [15,16,17,18,19], zeolites, silicas, porous organic-inorganic hybrid frameworks [20,21,22], and porous organic polymer networks [23,24,25], are excellent candidates for these applications. Among the porous organic systems, the Porous Aromatic Framework (PAF) family has received great attention recently [26,27,28], thanks to their exceptional stability and their very high surface area; in particular, PAF-302 (also called PAF-1 in the literature) exhibits one of the highest BET surface areas reported so far, along with a high affinity for methane [29,30,31], and to a certain extent, for CO_2_ [32,33,34,35,36].

A related, wide family of porous materials that have been investigated for decades is that of carbonaceous solids, obtained by carbonization of biomass [37,38,39] or by decomposition of organic materials, generally polymers [40], through physical or chemical activations [41,42,43]. Physical activation implies the pyrolysis of the precursor at high temperatures with gases such as carbon dioxide, steam, or others. Chemical activation also involves pyrolysis, but at lower temperatures, as well as impregnation of the starting precursor with chemicals such as H_3_PO_4_, ZnCl_2_, K_2_CO_3_, NaOH, or KOH [44,45,46,47,48]. Chemical activation offers several advantages with respect to the physical one, including an easy procedure, lower temperatures, shorter time, higher yields, and better porous structure of the resulting material [49,50,51].

In this field, a method which has provided excellent results is based on KOH activated carbonization at high temperature. This approach, applied to various microporous organic materials, led to porous carbons with surface area as high as 3000 m^2^ g^−1^ and excellent gas storage properties [23,52,53,54]. PAF-302 was also treated by KOH at different temperatures (500–900 °C), obtaining porous carbons with high surface area, high heat of adsorption, and very good uptakes of CO_2_, CH_4_, and H_2_ [55,56,57].

**Figure 1:**
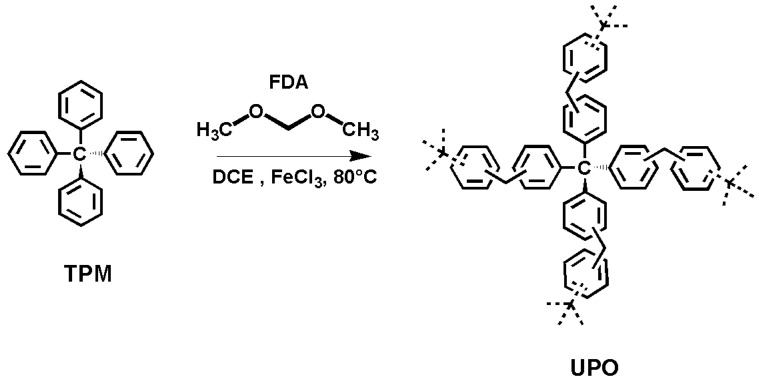
Scheme of the synthesis of UPO materials.

In our previous work, we reported on the synthesis and storage capacity of microporous solids with high surface area, belonging to the class of Hyper-Crosslinked Polymers (HCPs) [30]. In the following these solids are referred to as UPO (from University of Piemonte Orientale). UPO materials, prepared from tetraphenylmethane (TPM) and formaldehyde dimethyl acetal (FDA), represent a very attractive family of porous organic networks easily prepared by Friedel–Crafts reaction (Figure 1); in general, HCPs combine high gas uptake capacity, low synthetic costs, and easy industrial scalability [58,59,60,61,62,63].

**Figure 2:**
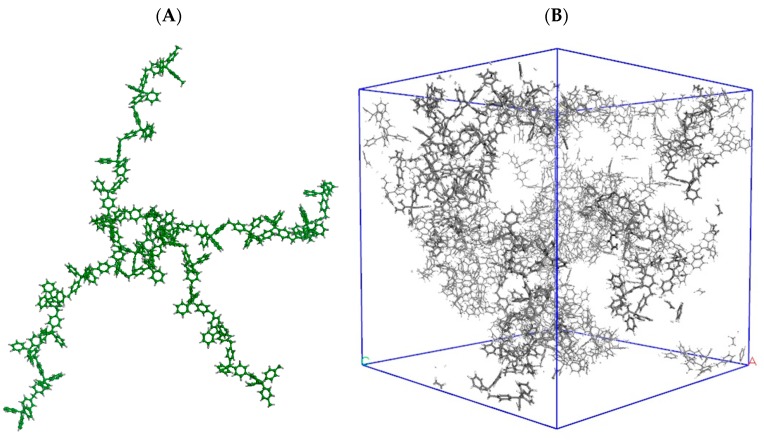
The building unit (**A**) and a possible 3D structure (**B**) of the model proposed for UPO materials in reference [60]. The building units are polymerized and assembled in disordered solids (**B**) with given macroscopic density.

Obtaining a reliable model of HCP amorphous structures is a very complex task. Recently, the UPO network was modeled by a trial-and-error procedure, based on the simulation of N_2_ and CO_2_ adsorption in different tentative models of suitable stoichiometry, compared with experimental uptake [64]. A snapshot of the structure proposed by this procedure is shown in Figure 2.

Herein, we report on the effect of different chemical activation and thermal treatments on UPO materials to obtain microporous carbons with improved surface area and total pore volume. Two polymers were used as precursors, namely UPO8 and UPO16, obtained by reacting different ratios of TPM and FDA (1:8 and 1:16, respectively); the post-synthesis treatments tested in this work include a mild thermal treatment at 380 °C, below the decomposition temperature, and three more aggressive treatments at 750 and 800 °C, combined with different activation reactions with KOH.

All the obtained carbon materials were carefully characterized, with different physico-chemical techniques. In particular, we described their textural properties (surface area, porous volume, pore size distribution) depending on the starting polymer and the post-synthesis treatment. The gas uptake performance at high pressure was then tested for the sample with the most interesting textural properties, and both gravimetric and volumetric storage of methane (298 K, up to 100 bar), carbon dioxide (298 K, up to 40 bar), and hydrogen (77 K, up to 100 bar) are reported. Furthermore different packing procedures were tested to form compact carbon samples from the pristine, as-synthesized powders.

## 2. Experimental Section

### 2.1. Synthesis of UPO Materials

The Hyper-Crosslinked Polymers were prepared using the procedure reported by some of the authors recently [30]; in particular, ferric chloride and TPM were suspended in dichloroethane (DCE, 135 mL). The resulting mixture was stirred vigorously at room temperature to obtain a homogeneous solution. Then, FDA was added drop-wise. The resulting thick gel was stirred at room temperature for 4 h and then heated under reflux overnight. After cooling to room temperature, the gel was diluted with ethanol and washed several times with water until the pH become neutral, and finally dried in oven at 100 °C overnight. The reaction was carried out using the TPM:FDA molar ratios of 1:8 (UPO8) and 1:16 (UPO16), while the FeCl_3_:FDA ratio was 1:1.

### 2.2. Post Synthesis Treatments

After the synthesis, the polymers were activated with the following thermal and chemical treatments.

(1) Heating activation at 380 °C: 1.0 g of UPO material was placed in an alumina crucible and heated to 380 °C for 16 h with a heating rate of 2 °C/min under nitrogen flow. The materials were labeled as UPO8-380 and UPO16-380, depending on the starting material.

(2) Chemical activation with KOH at high temperatures: a number of different chemical activation procedures were used, differing for the pre-activation method and the final temperature (800 or 750 °C), as detailed below.

Pre-activation method 1: 1.0 g of UPO material was homogeneously grinded with 3 g of KOH. The mixture was kept in the air for 5 h.

Pre-activation method 2: 1.0 g of UPO material was added to a 1 M solution of KOH in ethanol (95% *v/v* in water), with a 1:4 ratio by weight, and stirred for 18 h at room temperature. Subsequently, the mixture was dried under vacuum at 50 °C.

Pre-activation method 3: 1.0 g of UPO material was homogeneously grinded with 3 g of KOH, under inert conditions.

After the preparation, the mixture was placed in a crucible of alumina and thermally treated under N_2_ flow with a ramp of 2 °C/min up to 750 or to 800 °C, and subsequently held under isothermal conditions for 1 h. After the chemical activation, all the resulting carbons were washed with deionized water (250 mL), neutralized with 2 M HCl (200 mL), and washed again with deionized water to remove potassium salts, and then dried at 110 °C for 18 h.

The carbonaceous materials obtained by these procedures were labeled as KUPO*x-y-z*, where x indicates the FDA/TPM ratio of the parent polymer (i.e., 8 or 16), y refers to the pre-activation method (1, 2, or 3), and z to the activation temperature (750 or 800 °C). The yields of the polymers are between 45% and 55%. This discrepancy is related to the temperature activation process.

### 2.3. Characterization Techniques

The materials in KBr pellets were characterized by Fourier transform infrared spectroscopy (FT-IR) using a Bruker Equinox 55 spectrometer (Bruker Optics, Billerica, MA, USA) equipped with a pyroelectric detector (DTGS type) with a resolution of 4 cm^−1^. Raman spectra were recorded on as-prepared powders using a Jobin Yvon HR800 LabRam μ-spectrometer (Horiba, Kyoto, Japan), equipped with an Olympus BX41 microscope, a HeNe 20 mW laser working at 632.8 nm, and a charge-coupled device (CCD) air-cooled detector. Instrument calibration was carried out before each analysis, by checking the position and intensity of the Si band at 520.65 ± 0.05 cm^−1^. To improve the signal to noise ratio, 40 cycles of 70 s (about 11 h) were performed. The spectra were recorded in the 3500–500 cm^−1^ spectral region.

N_2_ physisorption measurements were carried out at 77 K in the relative pressure (*P*/*P*_0_) range 1 × 10^−7^ to 1 by using an Autosorb iQ/ASiQwin instrument (Anton Paar QuantaTec Inc., Boynton Beach, FL, USA). Prior to the analysis, the samples were outgassed at 150 °C for 16 h (residual pressure lower than 10^−6^ Torr). The apparent BET surface areas were calculated over a relative pressure range recommended by the “Micropore BET Assistant”, a program that is implemented in ASiQWin Quantachrome software to facilitate the selection points within the linear range of the BET plot for microporous materials. The pore size distribution for all samples is calculated using quenched solid density functional theory (QSDFT) on a carbon surface with slit/cylindrical geometry applied to the adsorption branch (method with the smallest fitting error).

Thermogravimetric analyses (TGA) were performed on a Setaram SETSYS Evolution instrument (SETARAM Instrumentation, Caluire, France) under Ar (gas flow rate 20 mL/min), heating the samples from 30 to 800 °C with a rate of 2 °C/min. The derivate of thermogravimetric curves (DTG) was also calculated.

The true (skeletal) density of the samples was measured by Helium pycnometry at room temperature. The apparent density of the as-synthesized powders was computed by combining the true density and the porous volume provided by N_2_ physisorption. Packing density was calculated by pressing the powder either to 0.75 or to 15 tons/cm^2^ for different times, as detailed in the following.

The EDS analyses were recorded on a Quanta 200 (FEI Company, Eindhoven, Netherlands) Scanning Electron Microscope equipped with energy dispersive spectrometer (EDAX Inc., Mahwah, NJ, USA) attachment, using a tungsten filament as electron source at 25 KeV

High-pressure gas adsorption measurements for H_2_, CO_2_, and CH_4_, were carried out at different temperature and pressure and the isotherms were performed on an automated Sieverts’ apparatus (PCT-Pro-E&E from SETARAM Instrumentation, Caluire, France). Prior to the gas adsorption measurements, approximately 200 mg of the sample were degassed at 150 °C under high vacuum for ~18 h. H_2_ adsorption measurements were carried out at liquid nitrogen temperature (77 K) up to a maximum pressure of 100 bar. Both CO_2_ and CH_4_ adsorption experiments were performed at room temperatures and up to a final pressure of 40 bar and 100 bar, respectively.

## 3. Results and Discussion

Two HPC polymers (UPO8 and UPO16), obtained by Friedel-Crafts reaction as described in reference [30] with 1:8 and 1:16 TPM/FDA ratios, respectively, were subjected to two post synthesis treatments, with the aim of improving their textural properties.

The polymers underwent either: (i) a thermal treatment at 380 °C, below the degradation temperature, with the purpose of clearing the polymer pores, or (ii) a chemical activation with KOH using differing pre-activation methods to find the best conditions for KOH moisture to diffuse into the UPO pores. The mixtures were then thermally treated at 750 or 800 °C.

### 3.1. Effects of Thermal Treatment at 380 °C

**Figure 3:**
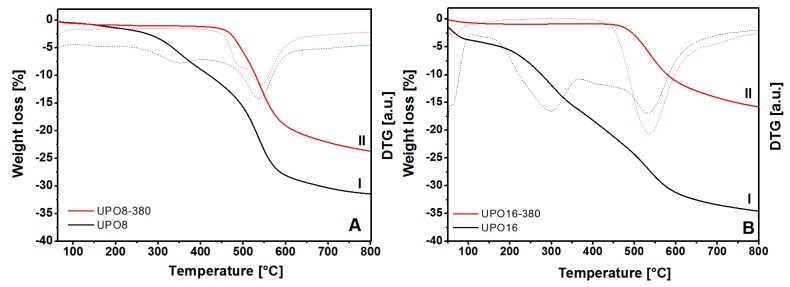
TGA analysis of UPO materials before (I) and after (II) thermal treatment at 380 °C for UPO8 (frame (**A**)) and UPO16 (frame (**B**)). The DTG derivatives are also reported (dashed curves). The analyses are performed under Ar flow (20 mL/min) using a heating ramp of 2 °C/min.

The results of the thermal gravimetric analysis (TGA) on the starting polymers and on the sample treated at 380 °C are compared in Figure 3.

The TGA profiles report two distinct weight losses in the original polymers (before the thermal treatment, curves I in Figure 3), one of which (centered at about 300 °C) is attributed to reaction impurities still present in the pores and the second (above 450 °C) to the framework decomposition. On the other hand, the thermal profiles of UPO8-380 and UPO16-380 samples (Figure 3, curves II) show only the weight loss starting at about 460 °C due to the structure decomposition. This behavior demonstrates the complete removal of impurities trapped in the porous structure upon treatment at 380 °C, however, this does not alter the thermal stability of UPO frameworks.

**Figure 4:**
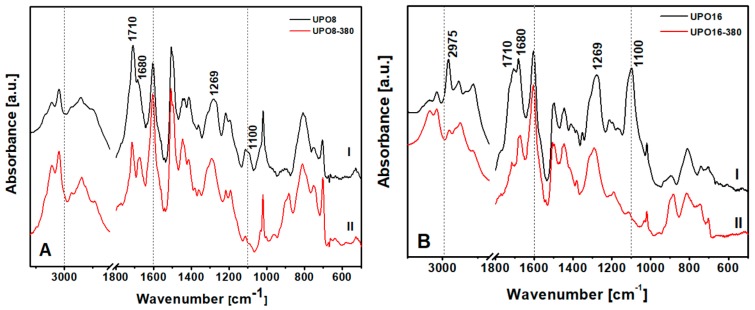
FTIR spectra in the region 3100–600 cm^−1^ of (**A**) UPO8 and (**B**) UPO16—samples before (I) and after (II) the thermal treatment at 380 °C.

To detect the type of impurities present in UPO8 and UPO16 starting materials, FTIR spectroscopy was used (Figure 4).

The bands between 3000 and 2800 cm^−1^, assigned to aliphatic C–H stretching, are strongly weakened after the thermal treatment (Figure 4, curves II: the effect is better appreciated by comparing them to the bands above 3000 cm^−1^, attributed to aromatic C–H stretching, left unaltered), demonstrating the removal of aliphatic impurities. In particular UPO16, synthesized with a higher FDA concentration, shows a band at 2975 cm^−1^ due to the asymmetric stretching of methyl groups in FDA fragments that disappears after thermal treatment. In the low frequency region, the bands at 1710 and 1680 cm^−1^, due to vibrations of substituted aromatic rings, undergo an intensity decrease probably caused by the removal of part of the chloro-methylene groups or FDA side-reaction products. Indeed, the band at 1269 cm^−1^, due to the CH_2_ wagging mode of the chloro-methylene groups, is still present, but with lower intensity compared to the situation before the treatment. Moreover, the absorption at 1100 cm^−1^, which is very intense in the starting polymers, disappears completely after treatment. This band has been assigned to ether groups and the disappearance confirms that the treatment cleaned the pores from trapped fragments of FDA. To summarize, the as-prepared UPO polymers show: (i) chloro-methylene groups attached to the aromatic rings of the polymer skeleton and (ii) FDA side-reaction products linked or trapped into the pore of the polymer. The precise nature of these fragments is far beyond the scope of the present paper, and will be the subject of a specific contribution.

**Figure 5:**
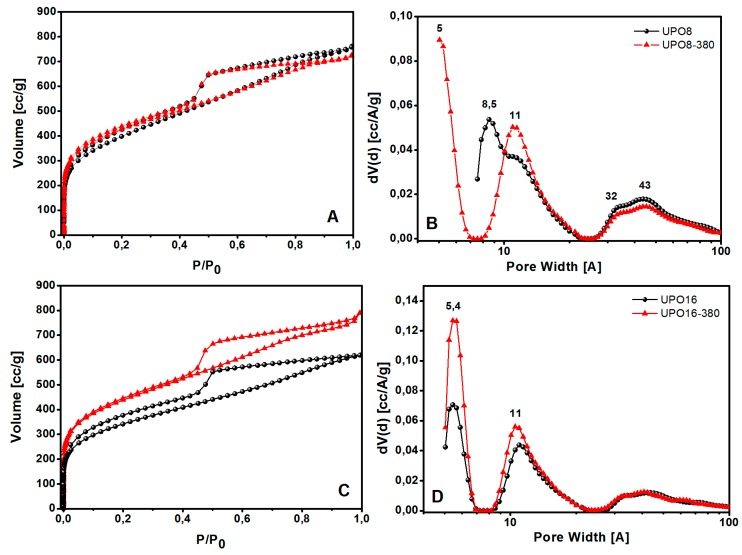
N_2_ adsorption-desorption isotherms at 77 K and pore size distributions of UPO polymers before (black circles) and after thermal treatment at 380 °C (red triangles): (**A**) UPO8 and UPO8-380 isotherms; (**B**) UPO8 and UPO8-380 PSD; (**C**) UPO16 and UPO16-380 isotherms; (**D**) UPO16 and UPO16-380 PSD.

Then the pore size distribution (PSD) of all the samples was analyzed by N_2_ physisorption at 77 K: the adsorption isotherms, and the corresponding PSD are reported in Figure 5 for UPO materials before and after the thermal treatment.

According to the IUPAC classification, all the isotherms shown in Figure 5 are of type I in the adsorption branch with H_2_-type hysteresis in the desorption branch. A large quantity of gas (>200 cm^3^/g) is adsorbed at low relative pressures, as expected for microporous materials; the isotherms also display a further gradual filling of mesopores at higher relative pressures in the range of 0.45–1 P/P_0_.

The porous properties of the polymers are summarized in Table 1. UPO8 shows a higher BET surface area (1435 m^2^∙g^−1^) than UPO16 (1289 m^2^∙g^−1^). After the thermal treatment, the surface area increases for both the materials, reaching a similar value higher than 1500 m^2^∙g^−1^. The increment of surface area is much larger for UPO16 (17%) than for UPO8 (6.5%), showing that the former sample contains a higher amount of side-products of the cross-linking polymerization entrapped inside the micropores, in agreement with TGA and FTIR results. Interestingly, while UPO16 and UPO16-380 exhibit a similar PSD with ultramicropores at about 5.4 Å and micropores at 11 Å, the effect of thermal treatment on UPO8 is different: in fact, ultramicropores at about 5.4 Å appear only after the thermal treatment, when micropores at 8.5 Å disappear and a family at 11 Å increases. In other words, the parent materials (UPO8 and UPO16) exhibit quite a different PSD, while their counterparts after the treatment (UPO8-380 and UPO16-380) are much more similar to each other. In all the cases, the thermal treatment at 380 °C led the micropore volume to increase, and the mesoporous volume to decrease.

The observed increase of the microporous volume upon thermal treatment is particularly interesting for gas storage or gas separation applications, as these kind of pores are responsible for the adsorption at low pressure.

### 3.2. Effects of Chemical Activation at High Temperature

UPO8 and UPO16 were also activated chemically with KOH at high temperatures, causing the carbonization of the materials: this is an effective method for the preparation of highly microporous materials. The activation mechanism occurs as a stoichoimetric solid–solid/solid–liquid reaction [63,64], according to equation 6KOH + 2C → 2 K + 3H_2_ + 2K_2_CO_3_. With the increase of the activation temperature above 700 °C, the resulting K_2_CO_3_ starts to decompose into K_2_O and CO_2_. Thus, the high microporosity in the carbon matrix is formed due to the ternary collaborative effects of chemical activation, physical activation, and the expansion of the carbon lattices by metallic potassium intercalation; this expansion is maintained even after the final wash.

The elemental composition of the KUPO materials was determined by EDX analysis, allowing exclusion of the presence of potassium in the washed samples.

In the literature [65] it is known that the key parameters for obtaining an efficient carbonization are the temperature, the nature of the activating agent, and the precursor/activating agent ratio. Here we investigated the effect of different combinations of these factors on the textural properties of the resulting carbons. Thus, UPO8 was either homogeneously grinded with KOH, and then left in open air for 5 h before carbonization (Method 1), or stirred for 18 h in a 1 M KOH solution in EtOH, and then dried and carbonized (Method 2), or grinded with KOH in inert atmosphere and then carbonized (Method 3). Since the carbonization temperatures were either 750 or 800 °C, a total of 6 porous carbon materials were obtain from UPO8; as for UPO16, for each activation method only the temperature that gave the best results for KUPO8 was applied, thus obtaining three KUPO16 materials in total.

**Figure 6:**
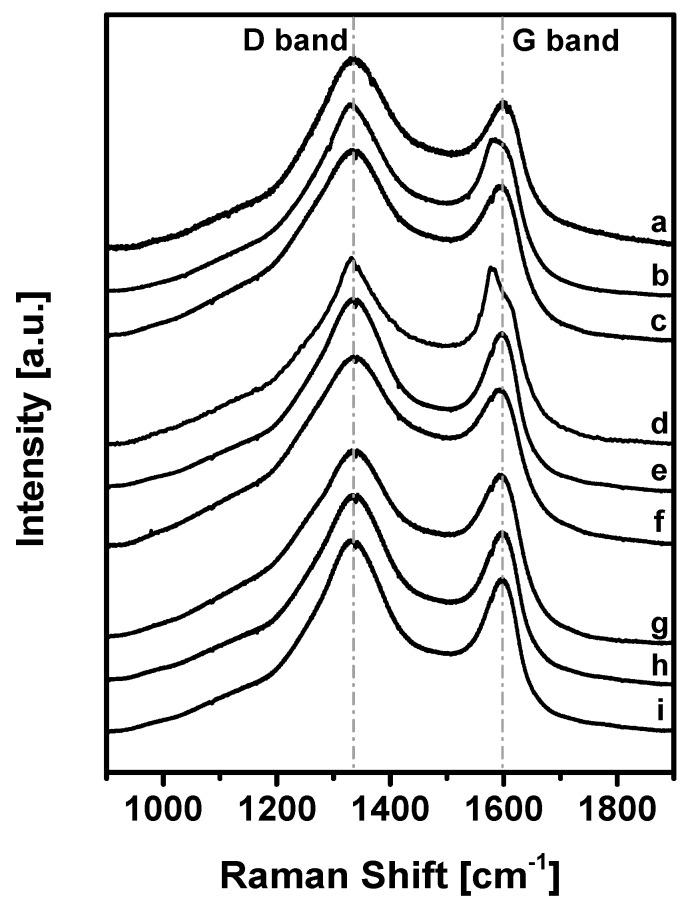
Raman spectra in the region 1900–900 cm^−1^ of KUPO carbon samples.

The carbonization degree after the KOH-activated thermal process described above was evaluated with Raman spectroscopy (Figure 6).

The curves correspond to the samples reported in Table 2.

The spectra show two main vibrational bands at 1340 and 1600 cm^−1^: the latter (G peak) corresponds to the Raman-allowed E2g mode in the ideal graphite, while the signal at 1340 cm^−1^ (D peak) corresponds to the disorder-induced band, which is associated with the large density of phonon states [66].

In particular, since the ratio of the bands D and G (*I*_D_/*I*_G_) is inversely related to the crystalline domain dimension [67], the strong D-peak in our samples demonstrates that the microporous carbons have an intermediate degree of graphitization (*I*_D_/*I*_G_ = 1.27–1.81) and contain a significant amount of disordered domains and defects.

Considering the three different carbonization methods, in all cases the value of *I*_D_/*I*_G_ decreases with the increase of temperature and the TPM/FDA ratio, indicating that these conditions lead to more ordered materials. Analogously, the *I*_D_/*I*_G_ values for the carbons obtained from UPO16 are systematically lower than those from UPO8, revealing again a more ordered structure for the former.

Compared to carbonized PAF-302 (*I*_D_/*I*_G_ = 0.70–0.90) [55], KUPO carbons have a higher *I*_D_/*I*_G_ ratio, and this can be explained by considering the structural difference of the parent materials. In HCPs, the –CH_2_– linkers make the structure more flexible and disordered than in PAF-302, also reflecting on the carbonized materials.

**Figure 7:**
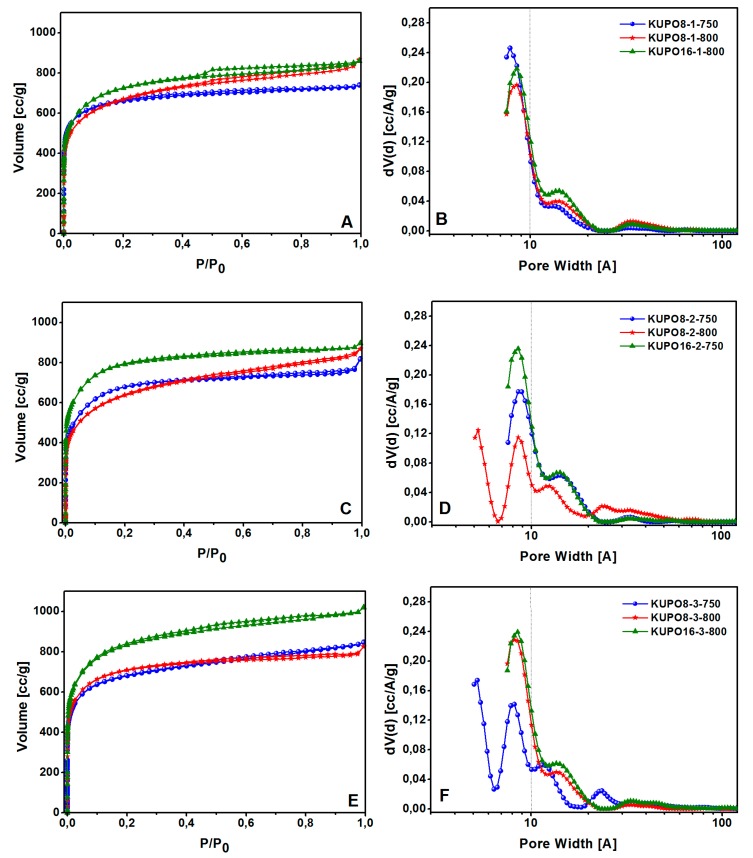
N_2_ adsorption-desorption isotherms at 77 K and PSD of KUPO carbons obtained with method 1 (**A**,**B**), method 2 (**C**,**D**), and method 3 (**E**,**F**), respectively.

The PSD and the pore volume were evaluated for KUPO materials with N_2_ physisorption at 77 K. The adsorption isotherms and the related pore size distributions are reported in Figure 7. The structural data were obtained from the adsorption branches by applying the QSDFT method, parameterized for carbon surfaces and slit/cylindrical pores (Table 3) [68].

All KUPO materials show a type I(b) isotherm with a minimal hysteresis at high relative pressures, and a large quantity of gas (>400 cm^3^∙g^−1^) adsorbed at low relative pressures, as expected for microporous materials. The carbonization of UPO16 with all three methods leads to similar PSD, with predominant micropores centered at 8.5 and 14 Å. On the contrary, the activated carbons obtained from UPO8 have different pore sizes depending on the activation method. Method 1 leads to a pore distribution centered at 8.5 and 14 Å (very similar to KUPO16), method 2 at 750 °C yields micro and mesopores, while at 800 °C ultramicropores at 5.2 Å are also formed; on the contrary, with method 3, micropores are obtained at 800 °C and both ultramicropores and micropores at 750 °C.

High surface areas, ranging from 2318 to 2975 cm^−1^, and high total pore volume, ranging from 1.03 to 1.30 cm^3^∙g^−1^, characterize all the activated carbons (Table 3). The apparent surface areas and pore volumes of the activated carbons are remarkably improved with respect to the parent materials.

It is noteworthy that all the results reported here refer to parent UPO materials not treated at 380 °C. If the same chemical and thermal activation procedures are applied to UPO8-380 or UPO16-380, the resulting carbons present systematically much lower surface areas and pore volumes (data not reported for simplicity). Thus, the removal of side-products obtained by heating at 380 °C with the consequent modifications of the textural properties described (see in Table 1) affects the carbonization process adversely.

The above data demonstrate that the chemical activation leads to porous carbons with improved textural properties, increasing microporous volumes and decreasing mesoporous volumes. The PSD and the total volumes are affected not only by the temperature but also by the pre-treatment with KOH, as well as by the textural and structural properties of the parent polymers. The pore occlusion, more abundant in the UPO16 sample, may in fact play a role in determining the higher pore volume and surface area of the carbon KUPO16.

Among the porous carbons obtained, KUPO16-2-750 is characterized by the highest surface area and microporous volume (Table 2), so we selected this material to test the gas storage capacity, measuring the excess adsorptions of CH_4_, H_2_, and CO_2_ at high pressures. The KUPO16-2-750 uptakes are compared to the results of the parent UPO16 to highlight the effect of the carbonization treatment.

### 3.3. High Pressure Gas Uptake

In particular, narrow pores (i.e., ultramicropores) are important for adsorbing gas at low pressures, since in these conditions the uptake is dominated by the host-guest interactions between gas molecules and pore walls. On the other hand, micropores and mesopores with small diameter, contributing to high surface area and large pore volume, are very important for the adsorption at higher pressure.

Note that the performance of an adsorbent in gas storage applications can be described either on gravimetric or on volumetric scales (measuring the quantity of adsorbed gas per gram or per cubic centimeter of adsorbent, respectively). Gravimetric uptakes are usually correlated to textural properties (surface area, porous volume, pore size distribution) [69,70,71], which are in turn often measured and reported per gram of material; on the other hand, volumetric uptakes also depend on the adsorbent density [72,73,74]. When different materials are compared, volumetric capacities are usually more reliable (if the densities are very different, less-dense materials are artificially favored in the gravimetric comparison); moreover, in practical applications, the main interest is often the amount of gas that can be stored in a reservoir of given volume.

In the following, the gravimetric gas uptakes of methane, carbon dioxide, and hydrogen are discussed first, then we consider how to effectively measure the density of compact samples to obtain a useful estimate of the volumetric uptakes.

For all the tested molecules, KUPO16-2-750 uptakes are highly improved with respect to UPO16, as expected due to the higher surface area and optimal porosity obtained under controlled KOH chemical activation. For CH_4_ storage, both the materials do not reach saturation at 100 bar, but the storage is still increasing with pressure. The maximum CH_4_ adsorption at 100 bar is 9 and 18 wt % for UPO16 and KUPO16-2-750, respectively.

**Figure 8:**
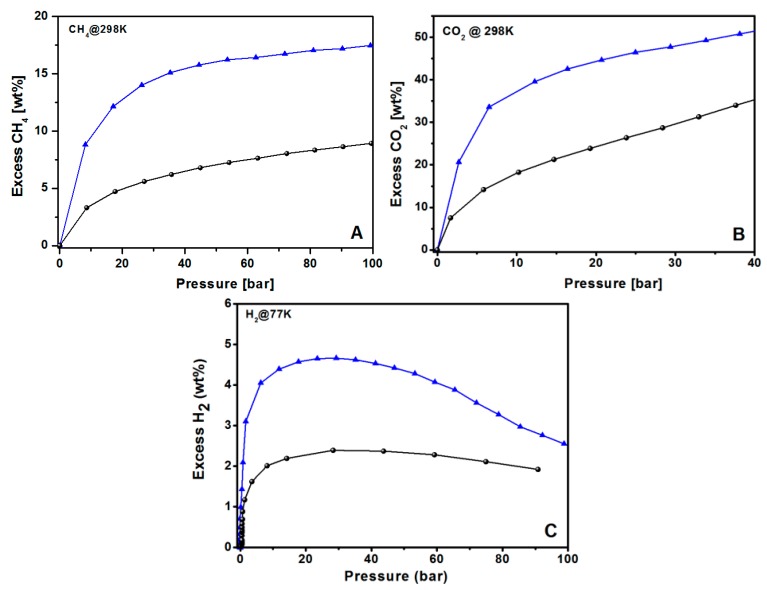
Gravimetric excess adsorption isotherms of UPO16 (black circles) and KUPO16-2-750 (blue triangles) for (**A**) methane (298 K, up to 100 bar), (**B**) carbon dioxide (298 K, up to 40 bar), and (**C**) hydrogen (77 K, up to 100 bar).

The CO_2_ uptake at 40 bar reaches 35 and 52 wt % (12,4 and 24,0 mmol/g) for UPO16 and KUPO16-2-750, respectively (Figure 8B). The excellent uptake of CO_2_ by KUPO16-2-750 is explained by the presence of high amounts of pores with sizes in the range 8–11 Å.

For both CO_2_ and CH_4_ storage, KUPO16-2-750 sorption properties compare well with those of the top performing carbon materials described in the literature (Table 4). For example, KUPO16-2-750 adsorbs 15.1 wt % of CH_4_ at 35 bar, whereas K-PAFs adsorb in the 8.7–17.1 wt % range [57], commercial activated carbons (ACs) (Maxsorb, F400, RGC30) in the 5.6–11 wt % range [8], ACs obtained from mesophase pitches in the 15.2–16.6 wt % range [8], and activated graphene-derived porous carbon in the 13.2–15.3 wt % range [75]. As for CO_2_ capture at 298 K and pressures up to 40 bar, the KUPO16-2-750 performance (52 wt %) is very high compared to other top performing carbons reported in the literature, namely K-PAFs (31.5–56.9 wt %) [57], commercial carbon AX21 (49.7 wt %, 20 bar) [76], and a-GDC graphene-derived carbons (41–48 wt %, 20 bar) [75].

Concerning the hydrogen adsorption, the maximum storage capacities of KUPO16-2-750 and UPO16 are 4.7 wt % and 2.2 wt % at 30 bar, respectively (Figure 8C). These data match well with the results from other porous carbon adsorbents with similar porosities. For example, Panella et al. [77] reported a capacity storage of 4.5 wt % at 77 K for the Activated Carbon I obtained from coke, whereas J. Wang et al. [78] reported H_2_ uptake of 4.2–4.7 wt % at 77 K for fungi-based porous carbons. H_2_ uptake of KUPO16-2-750 is also comparable (4.4–5.2 wt %) to different mesoporous carbide-derived carbons with very high surface areas (2500–3000 m^2^g^−1^) [79,80]. These results confirm the correlation of the hydrogen uptake capacity at high pressure with total micropore volume of ACs, with similar pore size and chemical surfaces [81].

### 3.4. Packing Densities

Both UPO and KUPO materials are synthesized as highly fine-grained powders: in this case, the apparent density estimated from the skeletal density (usually measured with helium adsorption) and the porous volume is often poorly related to the effective volumetric uptakes and to the storage capacity discussed below, for the presence of macropores and grain–grain mispacking.

Then KUPO16-2-750 powder was pressed into compact tablets that could be handled more easily, also eliminating the dead volume to a good extent (clearly this is different from the process of pellet or monolith formation, requiring more severe conditions). The samples were pressed at 0.75 tons/cm^2^ for variable times (from 10 to 180 min) until the density was stable; another sample was pressed at 15 tons/cm^2^ for 10 min. The resulting packing densities are reported in Table 5; a similar procedure with a pressure of 0.75 tons/cm^2^ was applied to measure the density and the gas storage of porous carbons in other studies [8,85].

After the compression, the tablet obtained at 15 tons/cm^2^ for 10 min (with the highest packing density) was further characterized with N_2_ adsorption to verify the extent to which the micro- and mesoporosity were affected, with the results illustrated in Table 6. The packing process cuts the total porous volume to 12%, entirely due to a small reduction of the microporous fraction.

### 3.5. Methane Storage Capacity of KUPO16-2-750

The performance of an adsorber in real working conditions is estimated better by the storage capacity (*n_stg_*), which corresponds to the density of gas that can be stored in a container completely filled by the adsorber. In other words, *n_stg_* is the absolute amount of gas per unit volume adsorbed inside the porous material plus the density of gas in the large macropores (which are not included in the porous volume measured by nitrogen) and in the interparticle space. To calculate this value, the following equation has been proposed [8]:

*n_stg_* = *n_exc_* + *r_gas_* (1 − *r_pack_*/*r_He_*)

combining the excess volumetric uptake (*n_exc_*) with the free gas density (*r_gas_*), the quantity in parentheses estimates the fraction of dead volume in the container from the adsorbent skeleton density (*r_He_*, determined by helium pycnometry) and its packing density (*r_pack_*).

**Figure 9:**
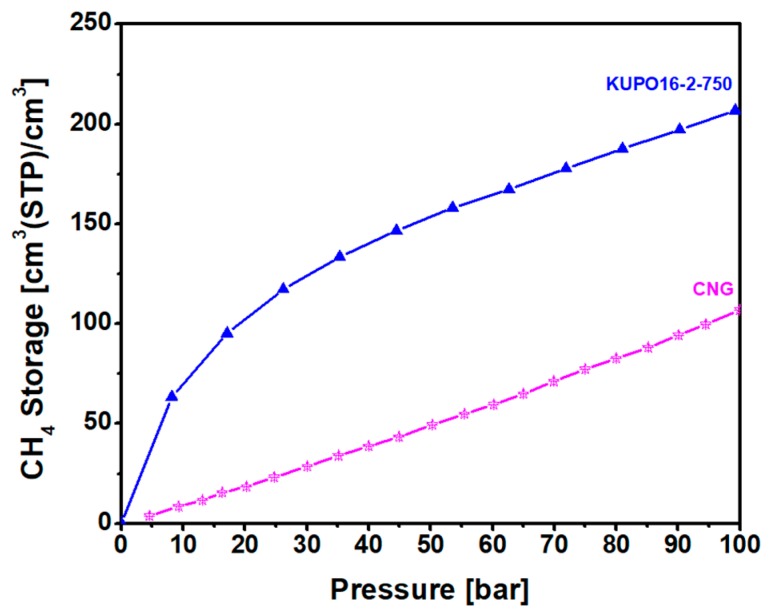
Methane storage capacity at 298 K of KUPO16-2-750 (blue triangles) compared to compressed methane gas (CG, pink stars).

The methane storage capacity of KUPO16-2-750 is illustrated in Figure 9 for pressures up to 100 bar. Considering the easy and low cost synthesis of the material, this performance is very satisfactory. At 20 bar, the storage capacity is almost equal to the capacity reported for LMA738, the best carbon material reported so far for this kind of application. For higher pressures, KUPO16-2-75 storage capacity is lower (210 *v/v* compared to 260 *v/v* for LMA738, at 100 bar) due to the smaller mesoporous volume. On the other hand, some widely-used commercial porous carbons (namely, F400 and Maxsorb) provide markedly lower storage capacities at 100 bar, at 166 and 210 *v*/*v*, respectively [8].

## 4. Conclusions

We have investigated different post-synthesis treatments applied to some microporous aromatic frameworks, whose preparation has been recently reported, to understand the structural modifications induced by the various treatments, and the possible applications for gas adsorption and storage.

Thermal treatment at 380 °C (i.e., below the polymer decomposition temperature) proved very effective in removing the side-products from the polymerization process: as a consequence, the treated UPO materials present higher BET areas, and increased microporous volume, along with a slight decrease of the mesoporous volume. Remarkably, the two samples tested here (and obtained with different precursors ratios) end up with very similar porosity after this treatment.

On the other hand, chemical treatment with KOH led to porous carbon materials with large microporous and mesoporous volume and BET area. Different pre-activation methods and temperatures were tested, finely tuning the textural properties. Such a tuning can be useful for developing materials for specific applications, e.g., KUPO8-3-750 seems more suited for gas separation at low pressure (due to the high micro- and ultramicroporous volume), while KUPO16-3-800 is expected to perform better for gas storage at higher pressure. It is also found that the best porous materials are obtained starting from parent UPO8 and UPO16 polymers. If the parent materials are previously subject to mild treatment at 380 °C to remove the reaction side products, the final porous carbons exhibit lower surface areas and porous volumes.

The gas uptake of the material with the highest surface area and microporous volume (KUPO16-2-750) was evaluated with high pressure adsorptions of methane, hydrogen, and carbon dioxide; the storage capacity, as recently defined, of KUPO16-2-750 with respect to methane was also measured and compared to the best performing carbon material described so far, with satisfactory results.

## Figures and Tables

**Table 1:** Textural properties (BET surface area and pore volume) for the samples before and after thermal treatment.

Sample	SSA BET (m^2^g^−1^) ^a^	Volume TOTAL (cm^3^g^−1^) ^b^	Volume MICRO (cm^3^g^−1^) ^c^			Volume MESO (cm^3^g^−1^) ^c^
			<7 Å	7 < Å< 20	Total	
**UPO8**	1435	1.09	--	0.31	0.31	0.78
**UPO8-380**	1528	1.04	0.14	0.27	0.41	0.63
**UPO16**	1289	0.96	0.09	0.22	0.31	0.65
**UPO16-380**	1513	0.99	0.14	0.28	0.42	0.57

^a^ Surface area calculated from N_2_ adsorption isotherm using the BET method; ^b^ Total pore volume at *P/P_0_* = 0.995; ^c^ Micropore volume derived using QSDFT method.

**Table 2:** I_D_/I_G_ values obtained from Raman spectra for all the KUPO carbons.

Curve	Sample	*I*_D_/*I*_G_
**a**	KUPO8-1-750	1.81
**b**	KUPO8-1-800	1.78
**c**	KUPO16-1-800	1.51
**d**	KUPO8-2-750	1.35
**e**	KUPO8-2-800	1.34
**f**	KUPO16-2-750	1.27
**g**	KUPO8-3-750	1.66
**h**	KUPO8-3-800	1.65
**i**	KUPO16-3-800	1.45

**Table 3:** Surface area and pore volume for KUPO materials compared to the parent polymers.

Sample	SSA_BET_ (m^2^g^−1^) ^a^	V_total_ (cm^3^g^−1^) ^b^	V_micro_ (cm^3^g^−1^) ^c^			V_meso_ (cm^3^g^−1^) ^c^
			<7 Å	7 < Å< 20	Total	
UPO8	1435	1.09	--	0.31	0.31	0.78
UPO16	1289	0.96	0.09	0.22	0.31	0.65
KUPO8-1-750	2500	1.03	--	0.93	0.93	0.11
KUPO8-1-800	2400	1.19	--	0.84	0.84	0.35
KUPO16-1-800	2700	1.20	--	0.98	0.98	0.22
KUPO8-2-750	2527	1.09	--	0.95	0.95	0.14
KUPO8-2-800	2318	1.21	0.14	0.50	0.64	0.57
KUPO16-2-750	2975	1.24	--	1.12	1.12	0.12
KUPO8-3-750	2562	1.20	0.24	0.54	0.78	0.42
KUPO8-3-800	2666	1.12	--	0.99	0.99	0.13
KUPO16-3-800	2950	1.30	--	1.07	1.07	0.23

^a^ Surface area calculated from the nitrogen adsorption isotherm using the BET method; ^b^ Total pore volume at *P/P*_0_ = 0.995; ^c^ Micropore and mesopore volumes derived using QSDFT method.

**Table 4:** Methane, hydrogen, and carbon dioxide uptake for different materials evaluated at different pressures and temperatures.

Material	CH_4_ Uptake, 298 K 35 bar (100 bar)	H_2_ Uptake, 77 K 30 bar	CO_2_ Uptake, 298 K 40 bar
	Wt %	Wt %	Wt %
KUPO16-2-750	15.1 (17.5)	4.7	51.3
K-PAFs [57]	8.7–17.1	3.5–6.2	31.5–56.9
GDC [75]	13.2–15.3	3.73–3.82 (10 bar)	41.0–48.0 (20 bar)
Maxsorb [8]	10.7 (12.3)	5.3 (40 bar) [79]	52.4 (50 bar) [82]
AX21	14.5 [83]	4.8 (40 bar) [84]	49.7 (20 bar) [76]

**Table 5:** Packing density (*r_pack_*) of KUPO16-2-750 samples pressed at different pressures for variable time.

Packing Pressure (tons cm^−2^)	Time (min)	*r_pack_* (g cm^−3^)
0.75	10	0.35
0.75	180	0.41
15.0	10	0.47

**Table 6:** BET surface area, total porous volume, micro- and mesoporous volumes of KUPO16-2-750 before and after the tablet formation (pressing at 15 tons/cm^2^ for 10 min).

Sample	SSA_BET_ (m^2^∙g^−1^)	V_total_ (cm^3^∙g^−1^)	V_micro_ (cm^3^∙g^−1^)	V_meso_ (cm^3^∙g^−1^)
As-synthesized	2975	1.24	1.12	0.12
After compression	2578	1.09	0.97	0.12

## References

[1] Deanna M.D., Berend S., Jeffrey R.L. (2010). Carbon Dioxide Capture: Prospects for New Materials. Angew. Chem. Int. Ed..

[2] Frota W.M., Sa J.A.S., Moraes S.S.B., Rocha B.R.P., Ismail K.A.R. (2010). Natural gas: The option for a sustainable development and energy in the state of Amazonas. Energy Policy.

[3] Jain I.P. (2009). Hydrogen the fuel for 21st century. Int. J. Hydrog. Energy.

[4] Kelly N.A., Gibson T.L., Cai M., Spearot J.A., Ouwerkerk D.B. (2010). Development of a renewable hydrogen economy: Optimization of existing technologies. Int. J. Hydrog. Energy.

[5] Matsuda R. (2013). Design and Synthesis of Porous Coordination Polymers Showing Unique Guest Adsorption Behaviors. Bull. Chem. Soc. Jpn..

[6] Broom D.P., Thomas K.M. (2013). Gas Adsorption by Nanoporous Materials: Future Applications and Experimental Challenges. MRS Bull..

[7] Marco-Lozar J.P., Kunowsky M., Carruthers J.D., Linares-Solano A. (2014). Gas Storage Scale-up At Room Temperature on High Density Carbon Materials. Carbon.

[8] Casco M.E., Martnez-Escandell M., Gadea-Ramos E., Kaneko K., Silvestre-Albero J., Rodriguez-Reinoso F. (2015). High-pressure Methane Storage in Porous Materials: Are Carbon Materials in the Pole Position?. Chem. Mater..

[9] Guerra G., Daniel C., Rizzo P., Tarallo O. (2012). Advanced Materials Based on Polymer Cocrystalline Forms. J. Polym. Sci. Part B Polym. Phys..

[10] Kim W.-G., Nair S. (2013). Membranes from Nanoporous 1D and 2D Materials: A Review of Opportunities Developments and Challenges. Chem. Eng. Sci..

[11] Ouyang Y., Shi H., Fu R., Wu D. (2013). Highly Monodisperse Microporous Polymeric and Carbonaceous Nanospheres with Multifunctional Properties. Sci. Rep..

[12] Zheng Y., Liu J., Liang J., Jaroniec M., Qiao S.Z. (2012). Graphitic Carbon Nitride Materials: Controllable Synthesis and Applications in Fuel Cells and Photocatalysis. Energy Environ. Sci..

[13] Rose M. (2014). Nanoporous Polymers: Bridging the Gap Between Molecular and Solid Catalysts. ChemCatChem.

[14] Pal N., Bhaumik A. (2015). Mesoporous Materials: Versatile Supports in Heterogeneous Catalysis for Liquid Phase Catalytic Transformations. RSC Adv..

[15] Kowalczyk P., Solarz L., Do D.D., Samborski A., MacElroy J.M.D. (2006). Nanoscale Tubular Vessels for Storage of Methane at Ambient Temperatures. Langmuir.

[16] Yang C.-M., Noguchi H., Murata K., Yudasaka M., Hashimoto A., Iijima S., Kaneko K. (2005). Highly Ultramicroporous Single-walled Carbon Nanohorn Assemblies. Adv. Mater..

[17] Linares-Solano A., Jordá-Beneyto M., Kunowsky M., Lozano-Castelló D., Suárez-García F., Cazorla-Amorós D., Terzyk A.P., Gauden P.A., Kowalczyk P. (2008). Hydrogen storage in carbon materials. Carbon Materials: Theory and Practice.

[18] Lim K.L., Kazemian H., Yaakob Z., Daud W.R.W. (2010). Solid-state Materials and Methods for Hydrogen Storage: A Critical Review. Chem. Eng. Technol..

[19] Marco-Lozar J.P., Kunowsky M., Surez-Garca F., Carruthers J.D., Linares A. (2012). Activated Carbon Monoliths for Gas Storage at Room Temperature. Energy Environ. Sci..

[20] Thomas K.M. (2009). Adsorption and Desorption of Hydrogen on Metal-organic Framework Materials for Storage Applications: Comparison with Other Nanoporous Materials. Dalton Trans..

[21] Marco-Lozar J.P., Juan-Juan J., Surez-Garca F., Cazorla-Amors D., Linares-Solano A. (2012). MOF-5 and Activated Carbons as Adsorbents for Gas Storage. Int. J. Hydrog. Energy.

[22] Peng Y., Krungleviciute V., Eryazici I., Hupp J.T., Farha O.K., Yildirim T. (2013). Methane Storage in Metal-organic Frameworks: Current Records Surprise Findings and Challenges. J. Am. Chem. Soc..

[23] Diaz U., Corma A. (2016). Ordered Covalent Organic Frameworks COFs and PAFs from Preparation to Application. Coord. Chem. Rev..

[24] Furukawa H., Yaghi O.M. (2009). Storage of Hydrogen Methane and Carbon Dioxide in Highly Porous Covalent Organic Frameworks for Clean Energy Applications. J. Am. Chem. Soc..

[25] Sang S.H., Furukawa H., Yaghi O.M., Goddard W.A. (2008). Covalent Organic Frameworks as Exceptional Hydrogen Storage Materials. J. Am. Chem. Soc..

[26] Ben T., Qiu S. (2013). Porous Aromatic Frameworks: Synthesis Structure and Functions. Cryst. Eng. Commun..

[27] Beaudoin D., Maris T., Wuest J.D. (2013). Constructing Monocrystalline Covalent Organic Networks By Polymerization. Nat. Chem..

[28] Pei C., Ben T., Qiu S. (2015). Great Prospects for PAF-1 and Its Derivatives. Mater. Horiz..

[29] Ben T., Pei C., Zhang D., Xu J., Deng F., Jing X., Qiu S. (2011). Gas Storage in Porous Aromatic Frameworks (PAFs). Energy Environ. Sci..

[30] Errahali M., Gatti G., Tei L., Paul G., Rolla G., Canti L., Fraccarollo A., Cossi M., Comotti A., Sozzani P. (2014). Microporous Hyper-crosslinked Aromatic Polymers Designed for Methane and Carbon Dioxide Adsorption. J. Phys. Chem. C.

[31] Ahmed A., Babarao R., Huang R., Medhekar N., Todd B.D., Hill M.R., Thornton A.W. (2015). Porous Aromatic Frameworks Impregnated with Lithiated Fullerenes for Natural Gas Purification. J. Phys. Chem. C.

[32] Babarao R., Dai S., Jiang D.-E. (2011). Functionalizing Porous Aromatic Frameworks with Polar Organic Groups for High-capacity and Selective CO_2_ Separation: A Molecular Simulation Study. Langmuir.

[33] Fu J., Wu J., Custelcean R., Jiang D.-E. (2015). Nitrogen-doped Porous Aromatic Frameworks for Enhanced CO_2_ Adsorption. J. Colloid Interface Sci..

[34] Fraccarollo A., Canti L., Marchese L., Cossi M. (2014). Monte Carlo Modeling of Carbon Dioxide Adsorption in Porous Aromatic Frameworks. Langmuir.

[35] Dhanalaxmi K., Yadav R., Kundu S.K., Reddy B.M., Amoli V., Sinha A.K., Mondal J. (2016). MnFe_2_O_4_ Nanocrystals Wrapped in a Porous Organic Polymer: A Designed Architecture for Water-Splitting Photocatalysis. Chem. Eur. J..

[36] Dhanalaxmi K., Singuru R., Mondal S., Bai L., Reddy B.M., Bhaumik A., Mondal J. (2017). Magnetic nanohybrid decorated porous organic polymer: Synergistic catalyst for high performance levulinic acid hydrogenation. ACS Sustain. Chem. Eng..

[37] Sun Y., Webley P.A. (2010). Preparation of activated carbons from corncob with large specific surface area by a variety of chemical activators and their application in gas storage. Chem. Eng. J..

[38] Krishnan K.A., Anirudhan T. (2003). Removal of cadmium(II) from aqueous solutions by steam-activated sulphurised carbon prepared from sugar-cane bagasse pith: Kinetics and equilibrium studies. Water.

[39] Crini G. (2006). Non-conventional low-cost adsorbents for dye removal: A review. Bioresour. Technol..

[40] Leonard A.D., Hudson J.L., Fan H., Booker R., Simpson L.J., O’Neill K.J., Parilla P.A., Heben M.J., Pasquali M., Kittrell C. (2009). Nano engineered carbon scaffolds for hydrogen storage. J. Am. Chem. Soc..

[41] Illan-Gomez M.J., Garcìa-Garcıìa A., Salinas-Martınez de Lecea C., Linares-Solano A. (1996). Activated Carbons from Spanish Coals 2: Chemical Activation. Energy Fuels.

[42] Bansal R.C., Donnet J.B., Stoeckli F. (1988). Active Carbon.

[43] Munoz-Guillena M.J., Illan-Gomez M.J., Martın-Martınez A., Salinas-Martınez de Lecea C. (1992). Activated carbons from Spanish coals 1 Two-stage CO_2_ activation. Energy Fuels.

[44] Joshi S., Adhikari M., Pokharel B.P., Pradhananga R.R. (2013). Effects of Activating Agents on the Activated Carbons Prepared from Lapsi Seed Stone. Res. J. Chem. Sci..

[45] Hameed B.H., Din A.T.M., Ahmed A.L. (2007). Adsorption of methylene blue onto bamboo-based activated carbon: Kinetics and equilibrium studies. J. Hazard. Mater..

[46] Badie S.G., Amina A.A., Nady A.F. (2007). Modification in adsorption characteristics of activated carbon produced by H_3_PO_4_ under flowing gases. Colloids Surf. A Phys. Chem. Eng. Asp..

[47] Onal Y., Akmil-Basar C., Sarici-Ozdemir C. (2007). Elucidation of the naproxen sodium adsorption onto activated carbon prepared from waste apricot: Kinetic equilibrium and thermodynamic characterization. J. Hazard. Mater..

[48] Ramakrishnan K., Namasivayam C. (2009). Development and Characteristics of Activated Carbons from Jatropha husk an Agro Industrial Solid Waste by Chemical Activation Methods. J. Environ. Eng. Manag..

[49] Yang K., Peng J., Srinivasakannan C., Zhang L., Xia H., Duan X. (2010). Preparation of high surface area activated carbon from coconut shells using microwave heating. Bioresour. Technol..

[50] Qiang Z., Gurkan B., Ma J., Liu X., Guo Y., Cakmak M., Cavicchi K.A., Vogt B.D. (2016). Roll-to-roll fabrication of high surface area mesoporous carbon with process-tunable pore texture for optimization of adsorption capacity of bulky organic dyes. Microporous Mesoporous Mater..

[51] Qiang Z., Liu X., Zou F., Cavicchi K.A., Zhu Y., Vogt B.D. (2017). Bimodal Porous Carbon-Silica Nanocomposites for Li-Ion Batteries. J. Phys. Chem. C.

[52] Hu Z., Srinivasan M.P. (1999). Preparation of High-surface-area Activated Carbons from Coconut Shell. Microporous Mesoporous Mater..

[53] Pandey S.S., Gerard M., Sharma A.L., Malhotra B.D. (2000). Thermal Analysis of Chemically Synthesized Polyemeraldine Base. J. Appl. Polym. Sci..

[54] Modak A., Bhaumik A. (2015). Porous Carbon Derived Via KOH Activation of a Hypercrosslinked Porous Organic Polymer for Efficient CO_2_ CH_4_ H_2_ Adsorptions and High CO_2_/N_2_ Selectivity. J. Solid State Chem..

[55] Zhang Y., Li B., Williams K., Gao W.-Y., Ma S.A. (2013). New Microporous Carbon Material Synthesized Via Thermolysis of a Porous Aromatic Framework Embedded with An Extra Carbon Source for Low-pressure CO_2_ Uptake. Chem. Commun..

[56] Ben T., Li Y., Zhu L., Zhang D., Cao D., Xiang Z., Yao X., Qiu S. (2012). Selective Adsorption of Carbon Dioxide by Carbonized Porous Aromatic Framework (PAF). Energy Environ. Sci..

[57] Li Y., Ben T., Zhang B., Fu Y., Qiu S. (2013). Ultrahigh Gas Storage Both at Low and High Pressures in KOH-activated Carbonized Porous Aromatic Frameworks. Sci. Rep..

[58] Dawson R., Stckel E., Holst J.R., Adams D.J., Cooper A.I. (2011). Microporous Organic Polymers for Carbon Dioxide Capture. Energy Environ. Sci..

[59] Dawson R., Ratvijitvech T., Corker M., Laybourn A., Khimyak Y.A., Cooper A.I., Adams D.J. (2012). Microporous Copolymers for Increased Gas Selectivity. Polym. Chem..

[60] Wood C.D., Bien T., Trewin A., Hongjun N., Bradshaw D., Rosseinsky M.J., Khimyak Y.Z., Campbell N.L., Kirk R., Stöckel E. (2007). Hydrogen Storage in Microporous Hypercrosslinked Organic Polymer Networks. Chem. Mater..

[61] Luo Y., Li B., Wang W., Wu K., Tan B. (2012). Hypercrosslinked Aromatic Heterocyclic Microporous Polymers: A New Class of Highly Selective CO_2_ Capturing Materials. Adv. Mater..

[62] Mondal J., Kundu S.K., Hung Ng W.K., Singuru R., Borah P., Hirao H., Zhao Y., Bhaumik A. (2015). Fabrication of Ruthenium Nanoparticles in Porous Organic Polymers: Towards Advanced Heterogeneous Catalytic Nanoreactors. Chem. Eur. J..

[63] Srinivasu P., Venkanna D., Kantam M.L., Tang J., Bhargava S.K., Aldalbahi A., Wu K.C., Yamauchi Y. (2015). Ordered hexagonal mesoporous aluminosilicates and their application in ligand-free synthesis of secondary amines. ChemCatChem.

[64] Canti L., Fraccarollo A., Gatti G., Errahali M., Marchese L., Cossi M. (2017). An atomistic model of a disordered nanoporous solid: Interplay between Monte Carlo simulations and gas adsorption experiments. AIP Adv..

[65] Lozano-Castello D., Calo J.M., Cazorla-Amoros D., Linares-Solano A. (2007). Carbon activation with KOH as explored by temperature programmed techniques and the effects of hydrogen. Carbon.

[66] Zhao J., Yang L., Li F., Yu R., Jin C. (2009). Structural evolution in the graphitization process of activated carbon by high-pressure sintering. Carbon.

[67] Tuinstra F., Koenig J.L. (1970). Raman spectrum of graphite. J. Chem. Phys..

[68] Neimark A.V., Lin Y., Ravikovitch P.I., Thommes M. (2009). Quenched Solid Density Functional Theory and Pore Size Analysis of Micro-mesoporous Carbons. Carbon.

[69] Yang S.J., Im J.H., Nishihara H., Jung H., Lee K., Kyotani T., Park C.R. (2012). General Relationship between Hydrogen Adsorption Capacities at 77 and 298 K and Pore Characteristics of the Porous Adsorbents. J. Phys. Chem. C.

[70] Presser V., McDonough J., Yeon S.-H., Gogotsi Y. (2011). Effect of pore size on carbon dioxide sorption by carbide derived carbon. Energy Environ. Sci..

[71] Jorda-Beneyto M., Suarez-Garcia F., Lozano-Castello D., Cazorla-Amoros D., Linares-Solano A. (2007). Hydrogen storage on chemically activated carbons and carbon nanomaterials at high pressures. Carbon.

[72] Yeon S.H., Knoke I., Gogotsi Y., Fischer J.E. (2010). Enhanced volumetric hydrogen and methane storage capacity of monolithic carbide-derived carbon. Microporous Mesoporous Mater..

[73] Jorda-Beneyto M., Lozano-Castello D., Suarez-Garcia F., Cazorla-Amoros D., Linares-Solano A. (2008). Advanced activated carbon monoliths and activated carbons for hydrogen storage. Microporous Mesoporous Mater..

[74] Zacharia R., Cossement D., Lafi L., Chahine R. (2010). Volumetric hydrogen sorption capacity of monoliths prepared by mechanical densification of MOF-177. J. Mater. Chem..

[75] Ganesan A., Shaijumon M.M. (2016). Activated graphene-derived porous carbon with exceptional gas adsorption properties. Microporous Mesoporous Mater..

[76] Coromina H.M., Walsh D.A., Mokaya R. (2016). Biomass-derived activated carbon with simultaneously enhanced CO_2_ uptake for both pre and post combustion capture applications. J. Mater. Chem. A.

[77] Panella B., Hirscher M., Roth S. (2005). Hydrogen adsorption in different carbon nanostructures. Carbon.

[78] Wang J., Senkovsk I., Kaskel S., Liu Q. (2014). Chemically activated fungi-based porous carbons for hydrogen storage. Carbon.

[79] Wang J., Oschatz M., Biemelt T., Borchardt L., Senkovska I., Lohe M.R., Kaskel S. (2012). Synthesis characterization and hydrogen storage capacities of hierarchical porous carbide derived carbon monolith. J. Mater. Chem..

[80] Kockrick E., Schrage C., Borchardt L., Klein N., Rose M., Senkovska I., Kaskel S. (2010). Ordered mesoporous carbide derived carbons for high pressure gas storage. Carbon.

[81] Wrobel-Iwaniec I., Dıez N., Gryglewicz G. (2015). Chitosan-based highly activated carbons for hydrogen storage. Int. J. Hydrog. Energy.

[82] Himeno S., Komatsu T., Fujita S. (2015). High-pressure adsorption equilibria of methane and carbon dioxide on several activated carbons. J. Chem. Eng. Data.

[83] Gandara F., Furukawa H., Lee S., Yaghi O.M. (2014). High Methane Storage Capacity in Aluminum Metal−Organic Frameworks. J. Am. Chem. Soc..

[84] Yang S.J., Jung H., Kim T., Park C.R. (2012). Recent advances in hydrogen storage technologies based on nanoporous carbon materials. Prog. Nat. Sci. Mater. Int..

[85] Casco M.E., Martinez-Escandell M., Gadea-Ramos E., Kaneko K., Silvestre-Albero J., Rodriguez-Reinoso F. (2015). Very high methane uptake on activated carbons prepared from mesophase pitch: A compromise between microporosity and bulk density. Carbon.

